# Maternal obesity alters endoplasmic reticulum homeostasis in offspring pancreas

**DOI:** 10.1007/s13105-016-0476-6

**Published:** 2016-03-15

**Authors:** Jumpei Soeda, Angelina Mouralidarane, Paul Cordero, Jiawei Li, Vi Nguyen, Rebeca Carter, Sabrina R. Kapur, Joaquim Pombo, Lucilla Poston, Paul D. Taylor, Manlio Vinciguerra, Jude A. Oben

**Affiliations:** Institute for Liver and Digestive Health, University College London, London, UK; Division of Women’s Health, King’s College London, London, UK; International Clinical Research Center (ICRC), Center for Translational Medicine (CTM), St. Anne’s University Hospital, Brno, Czech Republic; Centro Studi Fegato (CSF)-Liver Research Center, Fondazione Italiana Fegato, Trieste, Italy; Department of Gastroenterology and Hepatology, Guy’s and St Thomas’ Hospital, NHS Foundation Trust, London, UK

**Keywords:** Fatty pancreas, Obesity, Perinatal programming, ER stress, UPR, Autophagy

## Abstract

The prevalence of non-alcoholic fatty pancreas disease (NAFPD) is increasing in parallel with obesity rates. Stress-related alterations in endoplasmic reticulum (ER), such as the unfolded protein response (UPR), are associated with obesity. The aim of this study was to investigate ER imbalance in the pancreas of a mice model of adult and perinatal diet-induced obesity. Twenty female C57BL/6J mice were assigned to control (Con) or obesogenic (Ob) diets prior to and during pregnancy and lactation. Their offspring were weaned onto Con or Ob diets up to 6 months post-partum. Then, after sacrifice, plasma biochemical analyses, gene expression, and protein concentrations were measured in pancreata. Offspring of Ob-fed mice had significantly increased body weight (*p* < 0.001) and plasma leptin (*p* < 0.001) and decreased insulin (*p* < 0.01) levels. Maternal obesogenic diet decreased the total and phosphorylated Eif2α and increased spliced X-box binding protein 1 (XBP1). Pancreatic gene expression of downstream regulators of UPR (EDEM, homocysteine-responsive endoplasmic reticulum-resident (HERP), activating transcription factor 4 (ATF4), and C/EBP homologous protein (CHOP)) and autophagy-related proteins (LC3BI/LC3BII) were differently disrupted by obesogenic feeding in both mothers and offspring (from *p* < 0.1 to *p* < 0.001). Maternal obesity and Ob feeding in their offspring alter UPR in NAFPD, with involvement of proapoptotic and autophagy-related markers. Upstream and downstream regulators of PERK, IRE1α, and ATF6 pathways were affected differently following the obesogenic insults.

## Introduction

Obesity-associated non-alcoholic fatty pancreas disease (NAFPD) is a pathological condition characterized by ectopic fat deposition in the pancreas. In NAFPD, pancreatic stellate cells are abnormally activated, leading to excess collagen production and therefore pancreatic fibrosis [39]. This can potentially lead to pancreatic cancer [25], and currently, pancreatic adenocarcinoma is one of the deadliest malignancies in developed countries, with a prevalence increasing in parallel with obesity rates [7].

A possible cause of NAFPD is therefore obesity. Experimental models have demonstrated that maternal nutrition status during the perinatal period can directly modulate the risk of developing obesity [2], as well as obesity-associated metabolic diseases in offspring, such as non-alcoholic fatty liver disease (NAFLD) [3, 22], glucose intolerance [6], and hypertension [34]. Additionally, our research group has recently demonstrated that maternal obesity and subsequent obesogenic feeding in offspring can increase their triglyceride content and injury-related gene expressions in the pancreas [1].

Body and organ fat accumulation promotes the release of proinflammatory cytokines and increased oxidative stress, processes which might underlie the pathogenesis of both NAFLD [24] and NAFPD [5]. However, further studies focusing on other metabolic and molecular pathways are needed. It has been described that endoplasmic reticulum (ER) stress, a mechanism responsible for protein folding and maturation, is altered in obesity-related diseases [8]. External influences, such as nutrient supply, could cause disruption of ER homeostasis, resulting in an altered pattern of protein synthesis, also termed the unfolded protein response (UPR) [27]. ER stress-induced UPR activates three chief pathways, which are regulated by the following ER transmembrane proteins: the kinase RNA (PKR)-like ER kinase (PERK), the inositol-requiring 1 alpha protein (IRE1a), and the activating transcription factor 6 (ATF6). Activation of these proteins by phosphorylation or splicing initiates a cascade of reactions; consequently, they act as transcription modulators, activating downstream effectors and regulating the gene expressions [30]. UPR is primarily a protective pathway, but an unresolved UPR could lead to increased apoptotic-related mediators and cell death. Obesity is known to trigger UPR in the liver [12], and disruption of ER stress homeostasis is thought to contribute to apoptosis of hepatocytes, a feature in many end-stage liver diseases [18]. Since the pancreas is derived from the same embryonic origin as the liver [4], it is probable that NAFPD shares similar pathogenic mechanisms with NAFLD, involving ER stress. Therefore, by utilizing a developmental programming model, we studied if maternal obesity and a postweaning obesogenic diet could promote ER stress, thus affecting molecular pathways and cellular apoptosis in the pancreas, which in turn promote the pathogenesis of NAFPD.

## Materials and methods

### Animal models

Twenty female C57BL/6J outbred mice (Charles River Laboratories, Margate, UK) were randomly allocated to either a standard control diet (Con) (RM1, Special Dietary Services, Essex, UK) or an obesogenic high-fat-sucrose diet (Ob) (824053, Special Dietary Services) sweetened with condensed milk (Nestle, Vevey, Switzerland) as previously described [21]. The mice were kept on their respective diets 6 weeks before mating, during pregnancy and during lactation. Ob diet animals reached a 30 % increase in body weight before entering the mating cycle. The female animals were mated with male mice from the same litter in order to minimize genetic variability. Conception was determined by the formation of a vaginal plug. A total of four to five animals per experimental group gave birth (litters with at least five pups). Litters with five to nine pups were included into the study. Each litter was reduced to six pups within 48 h of birth. At day 21 postpartum, offspring were weaned onto either the control diet (Con) or the obesogenic diet (Ob) creating the following experimental groups: offspring of control weaned onto control diet (Con-Con), offspring of control weaned onto obesogenic diet (Con-Ob), offspring of obese weaned onto control diet (Ob-Con), and offspring of obese weaned onto obesogenic diet (Ob-Ob). At 6 months postpartum, female offspring were weighed, sacrificed by rising CO_2_, and the pancreata were extracted and appropriately weighed and stored for further analysis.

All the animals had access to food and water ad libitum and were kept in a thermostatically controlled environment (22 °C) in a 12-h light/dark cycle. The animals were treated in accordance with The Animal Scientific Procedures Act 1986 guidelines and UK Home Office. All the studies were approved by Local University College London Ethics Committee.

### Plasma biochemical analysis

Plasma samples were assayed for leptin (R&D 1301, BioVendor, Brno, Czech Republic) and insulin (90080, Crystal Chem, Downers Grove, IL, USA) using ELISAs, according to the manufacturers’ instructions.

### Western blotting

Western blotting analyses were performed as previously established [35]. Briefly, protein was isolated from murine whole pancreas tissue using RIPA lysis and extraction buffer (Thermo Scientific, Waltham, MA, USA) and quantified by the Pierce BCA Protein Assay Kit (Thermo Scientific). Thirty micrograms of protein per sample was loaded onto NuPAGE Bis-Tris Mini Gels (Life Technologies, Carlsbad, CA, USA) (*n* = 3–4 per experimental group) for electrophoresis and subsequently transferred to 0.45-μm Invitrolon PVDF membranes (Invitrogen, Life Technologies Corporation, UK) by electroblotting. The membranes were incubated in blocking buffer followed by overnight incubation with primary antibodies at 4 °C (Table 1); the membranes were then incubated with the secondary antibodies (Table 1). Finally, Pierce ECL Western Blotting Substrate (Thermo Scientific) was used for revelation procedures. The protein bands were visualized using FlourChem imager (ProteinSimple, CA, USA), and densitometry measurements were calculated using AlphaView SA software (version 3.4.0). The resulting bands were normalized against β-actin from the same samples.

**Table 1:** Western blot antibodies

Ab. against	Ab from	Company	Ref.	Dilution	MW (kDa)
p-PERK	Rabbit	Cell Signaling Technology	3179S	1:1000	170
PERK	Rabbit	Cell Signaling Technology	C33E10	1:1000	140
p-eIF2α	Rabbit	Cell Signaling Technology	9721S	1:1000	38
e-IF2α	Rabbit	Cell Signaling Technology	9722S	1:1000	30
p-IRE1α	Rabbit	Abcam	ab48187	1:1500	120
IRE1α	Rabbit	Cell Signaling Technology	14C10	1:1000	120
XBP1	Rabbit	Santa Cruz Biotechnology Inc	Sc-7160	1:2000	u-XBP1: 24–32 s-XBP1: 54–56
ATF6	Mouse	AbFrontier	70B1413.1	1:1000	p90-ATF6: 90 p50-ATF6: 50
GRP78	Rabbit	Cell Signaling Technology	3177	1:1000	78
CHOP	Mouse	Cell Signaling Technology	2895	1:1000	27
LC3B	Rabbit	Abcam	ab51520	1:1000	LC3BI:18 LC3BII: 16
β-Actin	Mouse	Santa Cruz Biotechnology Inc	Sc-47778	1:5000	42

### Quantitative real-time PCR

Pancreas samples were homogenized using TRIzol reagent (Invitrogen, CA, USA) by following the suppliers’ protocol. Sample concentrations were measured using a NanoDrop ND-1000 Spectrometer (Thermo Scientific). DNase treatment and retrotranscription to cDNA were carried out using the Qiagen QuantiTect Reverse Transcriptase kit (Qiagen). Quantitative real-time PCR (qPCR) was performed as previously described [28] (*n* = 3–4 per experimental group). GAPDH was used as a control housekeeping gene for all reactions. All primers were obtained from Sigma-Aldrich (San Luis, MO, USA) (Table 2). Ct values were calculated using the 2^−ΔΔCt^ method according to GAPDH as the internal control.

**Table 2:** Primers sequences and product sizes

Gene	Primer sequence	Product size
ATF4	Sense: GAGCTTCCTGAACAGCGAAGTG	113
	Antisense: TGGCCACCTCCAGATAGTCATC	
CHOP	Sense: TATCTCATCCCCAGGAAACG	219
	Antisense: GGGCACTGACCACTCTGTTT	
EDEM1	Sense: AGTCAAATGTGGATATGCTACGC	180
	Antisense: ACAGATATGATATGGCCCTCAGT	
HERP	Sense: GCAGTTGGAGTGTGAGTCG	229
	Antisense: TCTGTGGATTCAGCACCCTTT	
WSF1	Sense: CCATCAACATGCTCCCGTTC	64
	Antisense: GGGTAGGCCTCGCCATACA	
Insulin	Sense: AGCAAGCAGGTCATTGTTTCAA	96
	Antisense: AAGCCTGGGTGGGTTTGG	
18S	Sense: AGTCCCTGCCCTTTGTACACA	70
	Antisense: CGATCCGAGGGCCTCACTA	
GAPDH	Sense: TGAACGGGAAGCTCACTGG	307
	Antisense: TCCACCACCCTGTTGCTGTA	

### Statistical analysis

All data are shown as mean± standard error of the mean (SEM). Two-way ANOVA was applied for studying the effect of maternal obesogenic feeding and offspring obesogenic feeding. Comparison of the means was carried out by Tukey post hoc test. A statistically significant result was determined with a *p* value of less than 0.05. SPSS 14 software (SPSS, Chicago, IL, USA) was used for the statistical analysis.

## Results

### Effect of maternal and offspring obesogenic feeding in phenotypical and plasma biochemical parameters

**Fig. 1:**
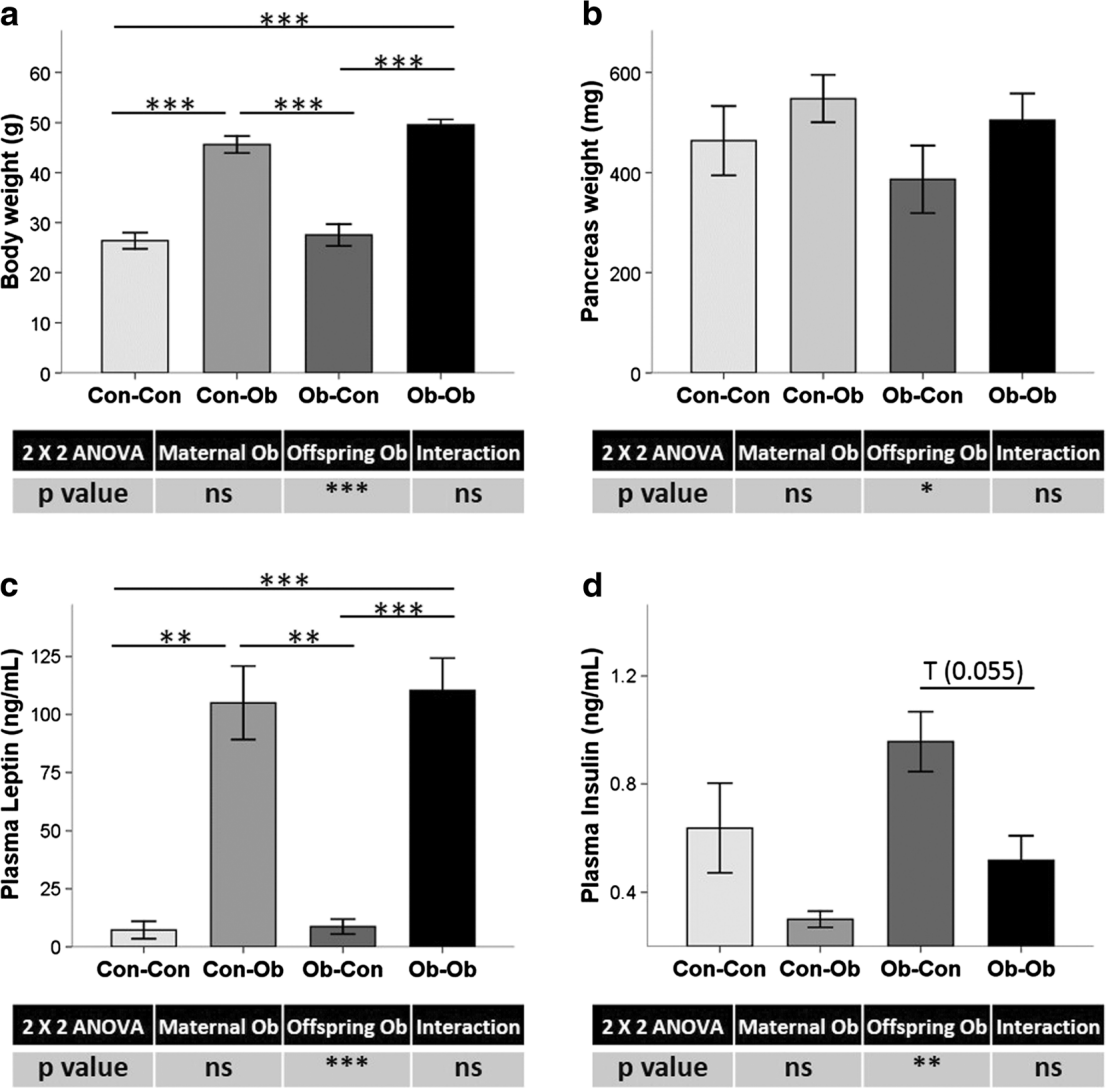
Phenotypic and biochemical parameters. Effects of maternal and offspring obesogenic feeding on **a** body weight, **b** pancreas weight, and plasma biochemical concentrations of **c** leptin and **d** insulin determined by ELISAs. *p < 0.05; **p < 0.01; ***p < 0.001; *n* = 4–5 animals per group

At 6 months old, offspring weaned onto an obesogenic diet had higher body weight (78 %, *p* < 0.001) (Fig. 1a) and pancreas weight (20 %, *p* < 0.05) (Fig. 1b), as compared with those weaned onto normal diets. Plasma leptin concentration had also increased due to offspring obesogenic diet (1275 %, *p* < 0.001) (Fig. 1c), while insulin concentrations decreased in comparison to control-fed offspring (−40 %, *p* < 0.01) (Fig. 1d).

We have previously described in this experimental model that maternal obesogenic feeding alone also predisposed offspring to a NAFPD phenotype, characterized by increased macrovesicular fat infiltration, worsened oral glucose tolerance test, and increased fibrogenic-related gene expression [1].

### Effect of maternal and offspring obesogenic feeding in pancreatic protein levels of UPR mainstream regulators

**Fig. 2:**
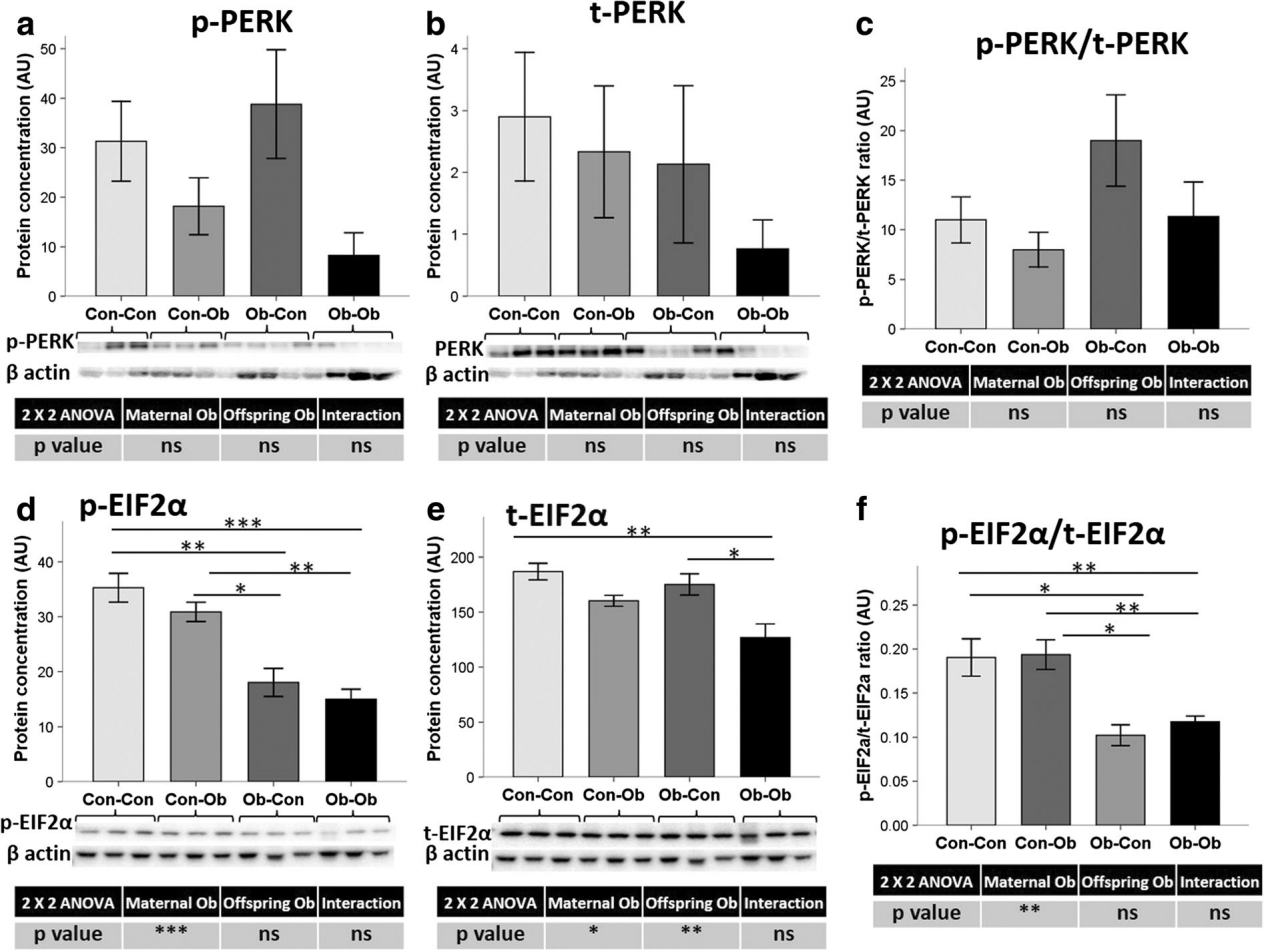
Pancreas protein concentration of PERK UPR pathway. Effect of maternal and offspring obesogenic feeding on **a** phosphorylated and **b** total PERK and **c** their ratio, as well as on **d** phosphorylated and **e** total EIF2α and **f** their ratio determined by Western blot. *p < 0.05; **p < 0.01; ***p < 0.001; *n* = 3–4 animals per group

UPR upstream regulator, PERK, did not show changes in protein level pattern by maternal or offspring obesogenic feeding, with an elevated intersample variability (Fig. 2a, b). However, UPR immediate activator eukaryotic initiation factor 2 alpha (EIF2α) total protein levels decreased in the offspring as an effect of maternal obesogenic feeding (−13 %, *p* < 0.05) (Fig. 2e), but also due to the direct obesogenic feeding in the offspring (−22 %, *p* < 0.01). Interestingly, the phosphorylation of EIF2α was significantly reduced by maternal obesity (−50 %, *p* < 0.001) (Fig. 2d), causing a significant decrease in the ratio between p-EIF2 α and t-EIF2 α (−42 %, *p* < 0.01) (Fig. 2f).

**Fig. 3:**
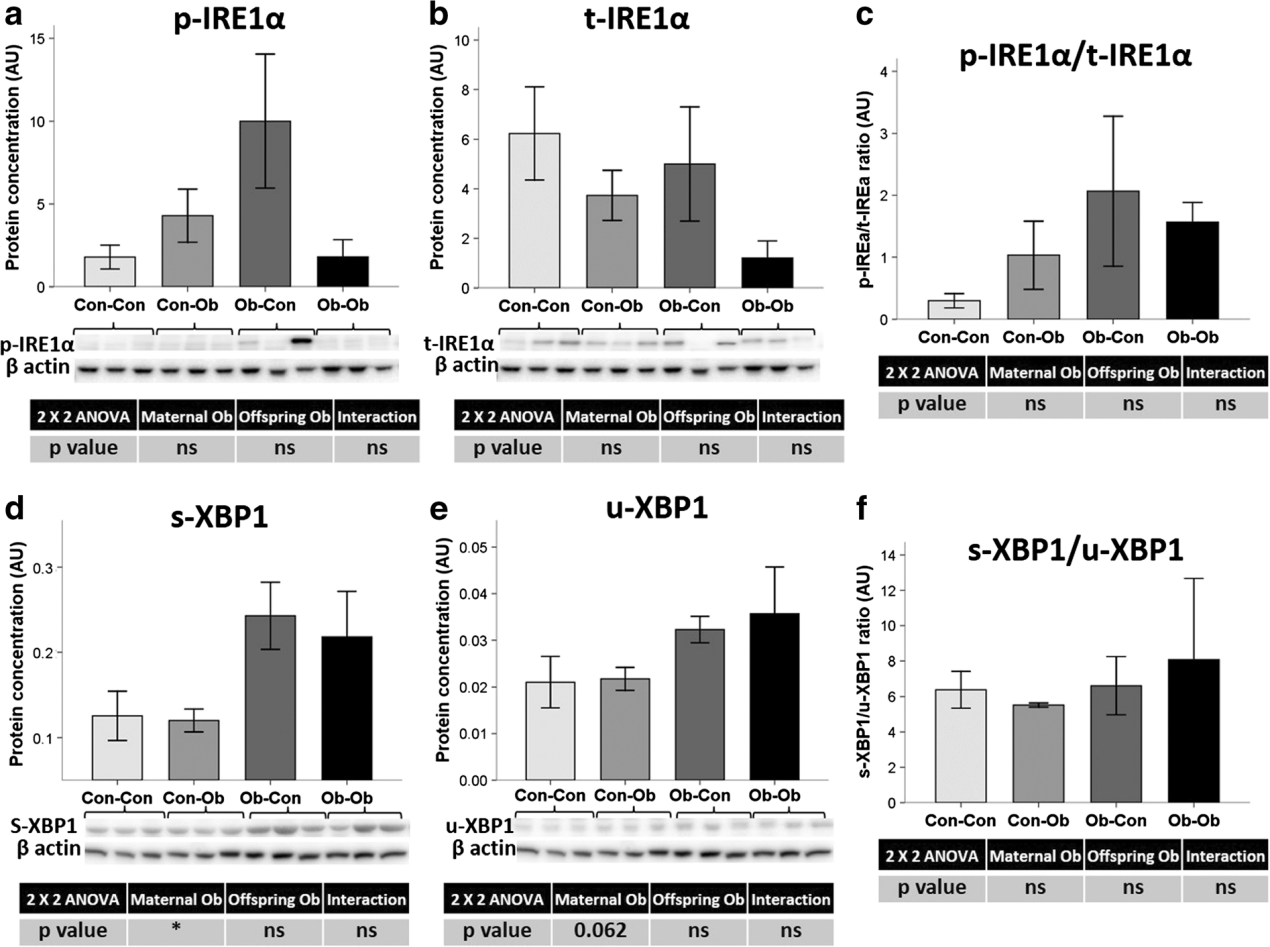
Pancreas protein concentration of IRE1α UPR pathway. Effect of maternal and obesogenic feeding on **a** phosphorylated IRE1α, **b** total IRE1α and **c** their ratio, as well as on **d** spliced XBP1 and **e** unspliced XBP1 and **f** their ratio determined by Western blot. *p < 0.05; *n* = 3–4 animals per group

According to the IRE1α branch, there was no statistical change observed in IRE1α protein concentrations, with no notable difference in phosphorylated or total hepatic protein levels, nor their ratio (Fig. 3a–c). However, maternal obesogenic feeding led to an increase in the levels of total unspliced and spliced X-box binding protein 1 (XBP1) (62 %, *p* < 0.1, and 87 %, *p* < 0.05, respectively) (Fig. 3d, e).

**Fig. 4:**
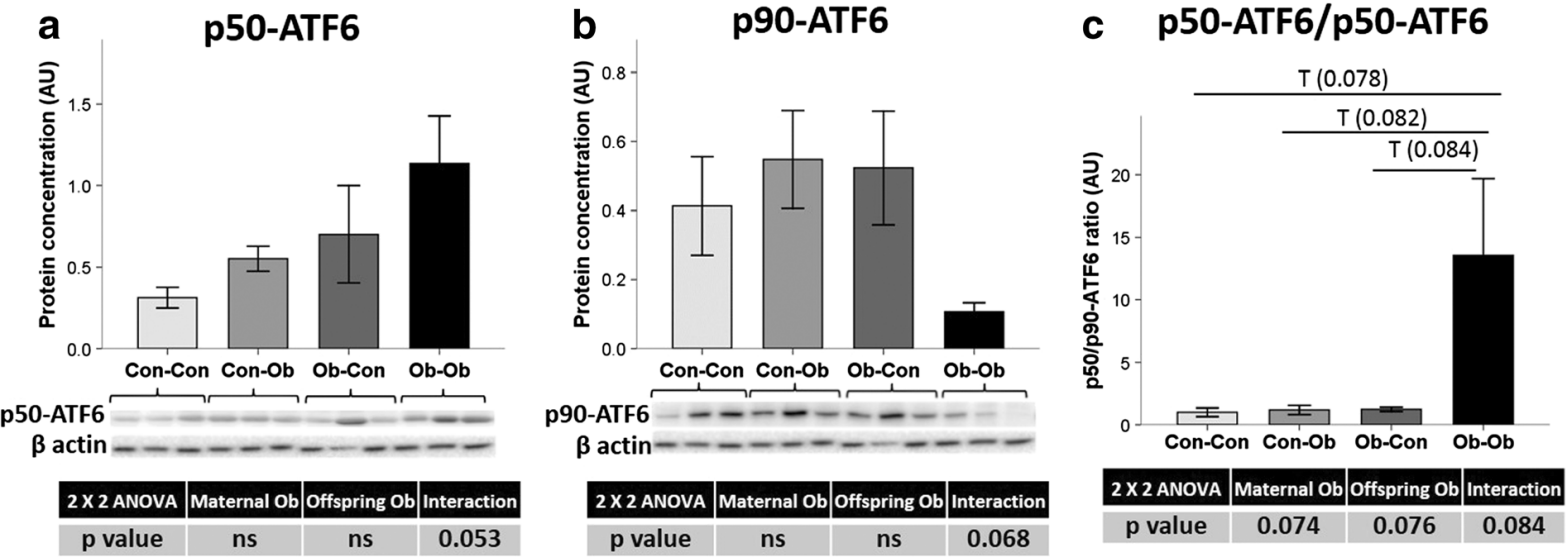
Pancreas protein concentration of ATF6 UPR pathway. Effect of maternal and offspring obesogenic feeding on **a** fragmented p50-ATF6, **b** p90-ATF6, and **c** their ratio determined by Western blot; *n* = 3–4 animals per group

For the ATF6 pathway, we observed an opposite tendency of interaction between maternal and offspring obesity in the whole protein (p90) and after splicing (p50) (Fig. 4a, b). This tendency was also reflected by the p50/p90-ATF6 ratio, which showed Ob-Ob animals with the highest ratio (from 1558 to 3437 %, *p* < 0.1) (Fig. 4c).

### Effect of maternal and offspring obesogenic feeding on pancreatic mRNA expression of other ER stress mediators

**Fig. 5:**
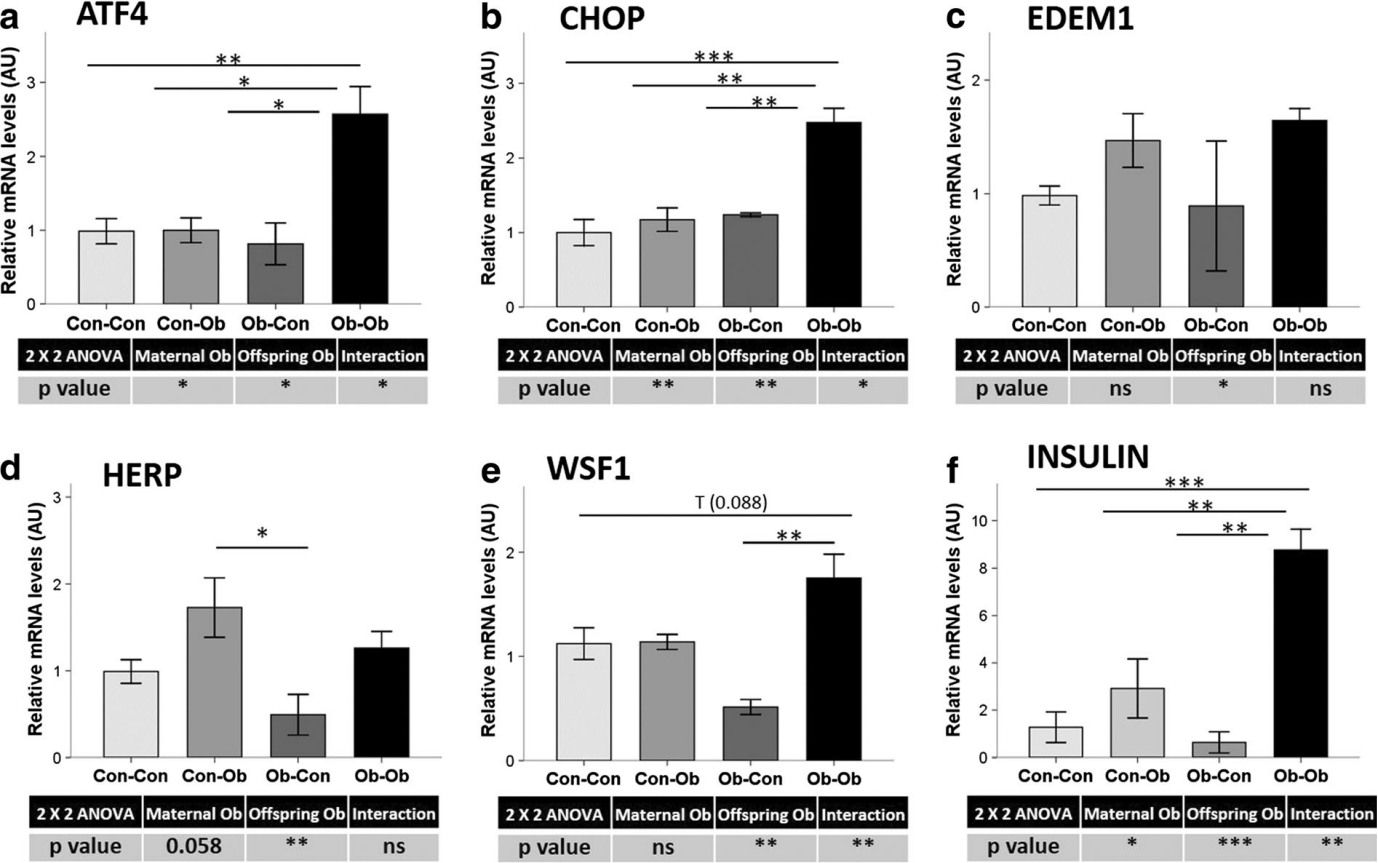
Pancreas mRNA levels by real-time qPCR. Effect of maternal and offspring obesogenic feeding on **a** ATF4, **b** CHOP, **c** EDEM, **d** HERP, **e** WSF1, and **f** insulin. *p < 0.05; **p < 0.01; ***p < 0.001; *n* = 3–4 animals per group

Activating transcription factor 4 (ATF4) and the proapoptotic transcription factor C/EBP homologous protein (CHOP) are mainly regulated by the PERK-eIF2α UPR pathway. Both maternal and offspring obesogenic feeding significantly increased their hepatic gene expression (*p* < 0.05 and <0.01, respectively) (Fig. 5a, b). Indeed, the “double hit” of obesogenic feeding in mother and offspring resulted in significantly increased Ob-Ob liver ATF4 and CHOP messenger RNA (mRNA) expressions in comparison with all other experimental groups (*p* from <0.05 to <0.01 and from 157 to 217 % for ATF4 and *p* from <0.01 to <0.001 and from 99 to 147 % for CHOP).

sXBP1-specific downstream target genes, ER degradation-enhancing alpha-mannosidase-like 1 (EDEM1), and homocysteine-responsive endoplasmic reticulum-resident (HERP) also increased their hepatic expression levels as an effect of obesogenic diets in offspring (66 %, *p* < 0.05 and 73 %, *p* < 0.01, respectively) (Fig. 5c, d). Maternal obesogenic feeding tended to decrease HERP expression levels (−19 %, *p* < 0.1). On the other hand, ER stress-related Wolfram syndrome 1 (WSF1) gene expression was increased by offspring obesogenic feeding (62 %, *p* < 0.01) (Fig. 5e), and comparisons between groups showed a tendency of WSF1 mRNA to increase in Ob-Ob compared with Con-Con mice (56 %, *p* < 0.1), as well as a significant increase in Ob-Ob compared with the Ob-Con group (243 %, *p* < 0.01).

Finally, hepatic insulin expression was increased due to obesogenic feeding in both mother and offspring (208 %, *p* < 0.05 and 491 %, *p* < 0.001, respectively) (Fig. 5f). The highest mRNA levels were observed in the Ob-Ob group, with significantly increased levels when compared with the all other groups (202 to 1294 %, *p* < 0.01 to <0.01).

### Effect of maternal and offspring obesogenic feeding on pancreatic protein levels of CHOP and GRP78

**Fig. 6:**
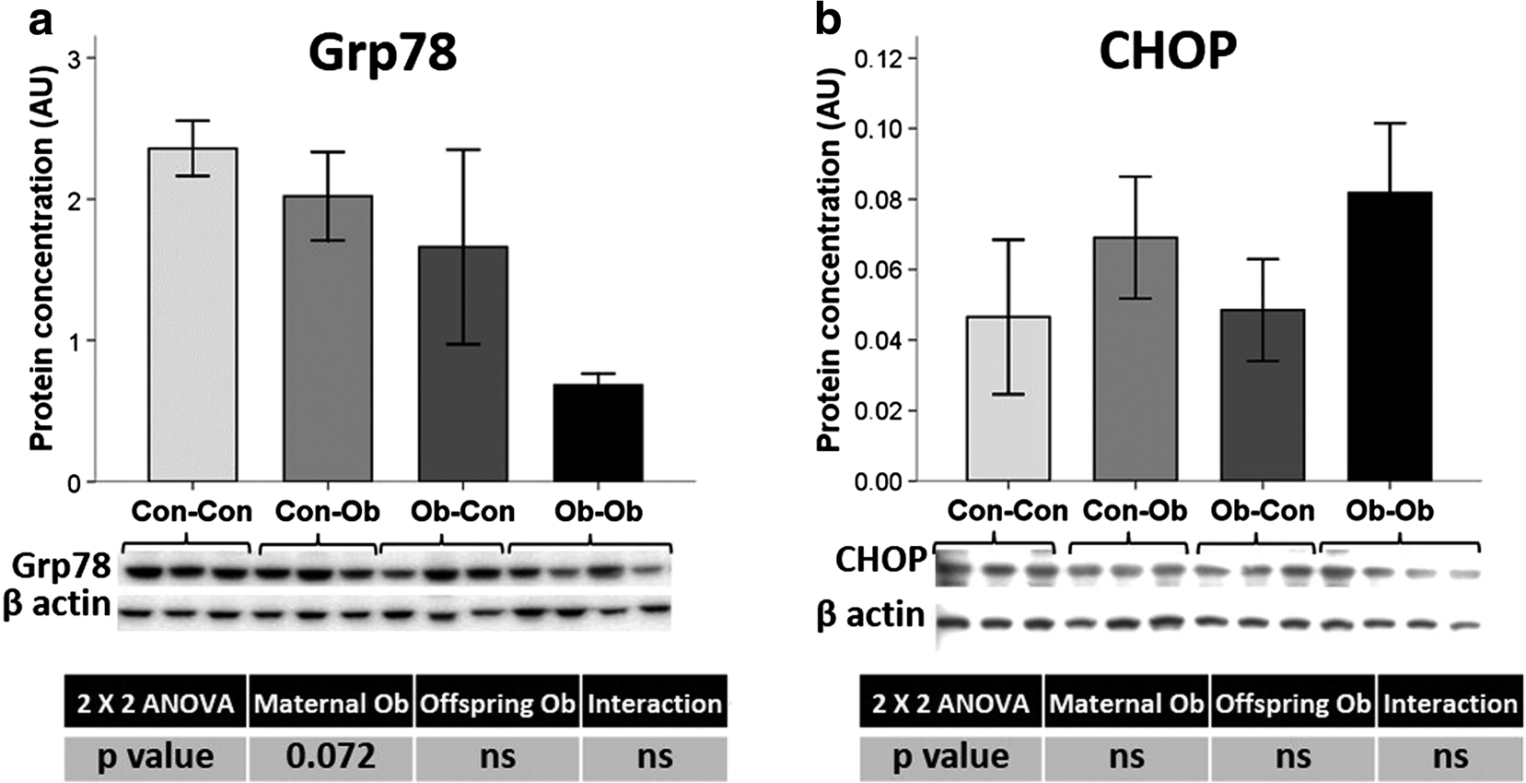
Pancreas protein concentration. Effects of maternal and offspring obesogenic feeding on **a** GRP78 and **b** CHOP determined by Western blot; *n* = 3–4 animals per group

GRP78 is a chaperone and master regulator of ER homeostasis. It inactivates the transmembrane PERK, IREα, and ATF6 blocking the UPR [30]. GRP78 levels showed a tendency to decrease in the groups with maternal obesogenic feeding (−43 %, *p* < 0.1) (Fig. 6a); conversely, results for CHOP protein concentrations did not show any statistically significant differences (Fig. 6b).

### Effect of maternal and offspring obesogenic feeding on autophagy regulators

**Fig. 7:**
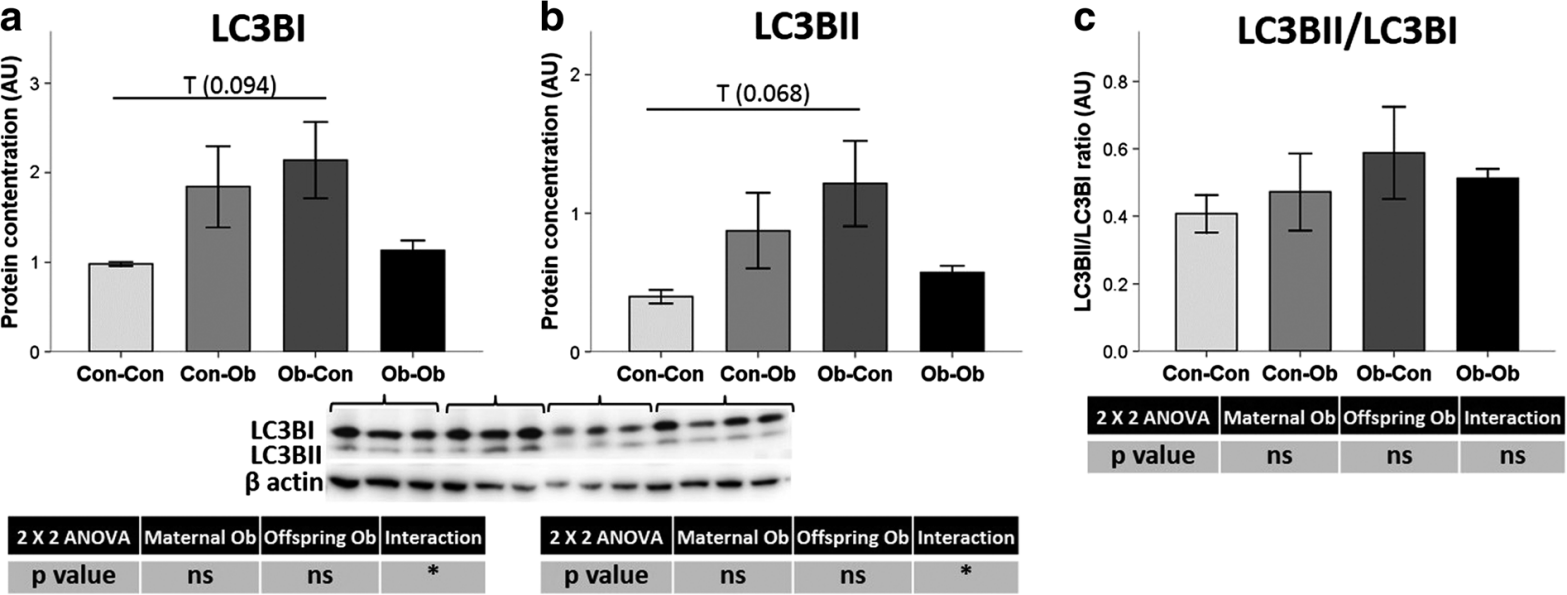
Pancreas protein concentration. Effects of maternal and offspring obesogenic feeding on the autophagy-related protein **a** LC3BI, **b** LC3BII, and **c** their ratio. *p < 0.05; *n* = 3–4 animals per group

NAFPD and pancreatic carcinogenesis are kept going by derangement of proliferation, apoptosis, metabolism, and autophagy [33]. Autophagy markers, microtubule-associated protein 1 light chain 3 beta (LC3BI and LC3BII), were quantified as possible mechanisms that could be modified by the dysregulation of ER homeostasis (Fig. 7a, b). We observed a trend toward increased levels of LC3BI and LC3BII protein levels due to maternal obesogenic feeding when compared with Con-Con and Ob-Con (118 and 205 %, respectively, *p* < 0.1 for both). However, there was no statistically significant difference in LC3BI/LC3BII ratios (Fig. 7c).

## Discussion

Our programming model has previously demonstrated that a NAFPD phenotype can be induced by maternal and offspring obesogenic feeding [1]. In this study, we show that ER stress plays a role in the pathogenesis of NAFPD and that this effect is a consequence of developmentally programmed obesity. To our knowledge, this is the first study exploring ER stress as a potential mechanism of developmental programming and NAFPD. It has been suggested that inhibiting ER stress may reduce the complications of obesity [10]. If ER stress is involved in the pathogenesis of NAFPD, it could similarly serve as a potential therapeutic target for pancreatic injury.

Similar to previous rodent models, our findings demonstrate that a mouse model of high-fat-diet-induced obesity can result in a pancreatic phenotype similar to that seen in human NAFPD: fat deposition in the pancreas, increased total body fat content, an obesity-associated physiological and biochemical profile, increased cytokine production, and, in some cases, even pancreatic cirrhosis, which in turn could lead to pancreatic cancer [5, 20, 25].

Novel evidence suggested that ER stress plays an important role in the pathogenesis of obesity and its related comorbidities [13]. Therefore, we initially expected to found an upregulation of ER stress in those experimental groups where offspring were obese. Our study has further discovered that the three major UPR pathways are differentially activated by maternal obesity, by post-weaning obesogenic diet, or by the combination of both factors. When PERK is activated by ER stress, eIF2α is phosphorylated which reversibly attenuates protein translation in the ER to reduce the overload of protein folding [30]. These findings are consistent with those of Wang et al. demonstrating that the reduction of PERK pathway gene expression significantly affects β-cell function and glucose homeostasis in a transgenic mouse model [36]. Similarly, Kennedy and colleagues showed that PERK and eIF2α are downregulated when serum glucose levels were high [11]. Another study involving rat pancreas damaged by ethanol also showed an increase in sXBP1, which is again similar to our findings, although unspliced protein did not show an increase in this setting [16].

The UPR pathway involving ATF6 is particularly important for regulating GRP78, XBP1, and CHOP expression [32]. After dissociating from GRP78, ATF6 as p90-ATF6 form is usually moved to the Golgi organelle to produce its active form, p50-ATF6. The active form is then translocated to the nucleus, resulting in upregulation of UPR processes, i.e., an increase in protein folding, processing, and degradation [30]. According to our results, although not statistically, maternal and offspring obesogenic feeding appeared to have a significant impact on ATF6 activation. This tendency drives to the hypothesis that ER stress increases in obesity situation. Furthermore, p50-ATF6 is known to activate CHOP transcription, which was also confirmed in the Ob-Ob group in our study.

WSF1 is an ER transmembrane protein with genetic polymorphisms that have been linked to the concurrent decrease in plasma insulin concentrations [29]. Thus, the marked WSF1 expression increase observed by the obesogenic feeding may account for the concurrent decrease in plasma insulin levels and greater pancreatic injury in the obesogenic feeding groups.

ER stress causes molecular chaperone GRP78 dissociation from the main ER stress sensors, activating the UPR [30]. Thus, transcriptional activation of GRP78 is used to determine whether there is an ER stress response. Although there was no statistically significant difference, protein expression levels of GRP78 were unexpectedly downregulated in Ob-Ob animals. Interestingly, a decrease on GRP78 liver expression has been found in db/db diabetic mice, which may be associated with pancreatic regulation of insulin resistance [37]. It is unclear why GRP78 was downregulated, even though ER stress was apparent, as shown by the upregulation of the ATF6 pathway. Liu et al. investigated the role of GRP78 in acute pancreatitis and found that downregulation of GRP78 was associated with increased apoptosis [15]. Therefore, the three pathways might regulate the action of the proapoptotic CHOP protein by upregulation during severe or chronic ER stress [14]. Furthermore, overexpression of CHOP leads to cell cycle arrest or apoptosis [23]. The implication of CHOP in the apoptotic pathway resulting from ER stress suggests that CHOP is a crucial link between hepatic fat infiltration and ER stress [19]. Another study, based on a chemically induced pancreatic ER stress rat model, had increased eIF2α phosphorylation and CHOP expression with no changes on GRP78. This pathological pancreatic condition was associated with an increased autophagy as a rescue system to maintain cytoplasmic homeostasis [9].

One possible mechanism for controlling the excess of misfolded proteins and cellular apoptosis is autophagy [17]. Autophagy controls the quality and homeostasis of the cytoplasm by eliminating unwanted proteins and damaged organelles by digestion in the lysosome [31]. Interestingly, autophagy has been suggested as a potential regulatory mechanism for insulin resistance following ER stress in diabetes [38]. Nonetheless, autophagy can also cause apoptosis [40]. Once the cytosolic form from LC3BI is cleaved by ATG4, it is conjugated with phosphatidylethanolamine and as LC3BII form act on autophagosome membrane promoting its expansion [26]. A double obesogenic hit resulted in low levels of LC3BI and LC3BII and in a trend toward an increase of Ob-Ob with respect to the control animals, with no changes in either parameter in the Ob-Con group. Surprisingly, these results were not in line with the previously described UPR activation profile. A potential explanation could be that circadian rhythmicity in the pancreas activates different metabolic regulators at different time points [1]. In support of this, preliminary results (unpublished) from our group have demonstrated that maternal obesogenic feeding and offspring feeding alter 24-h rhythmicity in NAFLD-associated ER stress homeostasis in mice, suggesting that the main regulators may impinge on different activation patterns at different times of the day.

Although we tried to control all the possible confounding factors in this transgenerational model, there could be some factors that limit this study, such as the genetic background of the dams and their fertility rate. Other limitation could be the lack of data from male offspring or the limited number of samples, which affected statistical results during the analyses due to the wider dispersion of the data. Finally, it is difficult to extrapolate the results of this experimental model to humans.

In summary, we have demonstrated that maternal obesity and postnatal obesogenic diets can induce an ER stress unbalance and result in a NAFPD phenotype with more severe associated metabolic outcomes. This is likely related to upregulation of ER stress pathways with resultant inflammation and cellular apoptosis, as suggested by increased proapoptotic and autophagy markers observed. However, while some UPR pathways appeared to be upregulated, these findings were not consistent across all the ER stress pathways, suggesting that while UPR dysregulation plays a role, ER homeostasis and fatty pancreas pathogenesis are regulated by other important factors. Further research in this area is therefore warranted, given the potential of these new therapeutic targets for the management of many obesity-associated diseases, including NAFPD.
